# Phytochemicals of Roman chamomile: Antioxidant, anti-aging, and whitening activities of distillation residues

**DOI:** 10.1515/biol-2025-1177

**Published:** 2025-10-13

**Authors:** Liyuan Sui, Ying Wang, Xiangyu Zhang, Min Yuan, Hong Ju, Jinlian Li

**Affiliations:** College of Pharmacy, Jiamusi University, Jiamusi, 154007, Heilongjiang, China; Jiamusi University Hospital, Jiamusi, 154007, Heilongjiang, China

**Keywords:** Roman chamomile, residue, antioxidant, antiaging, whitening

## Abstract

The large demand for Roman chamomile essential oil leads to nonnegligible residues in the process of steam distillation. It is an urgent problem to recycle these residues to solve the pollution in the ecological environment and enhance the industrial value. In this study, the components of different fractions extracted from the Roman chamomile residue were analyzed, and their antioxidant, whitening, and anti-aging activities were evaluated. It was found that the crude extract (CE) contained large amounts of polyphenols and flavonoids and displayed obvious antioxidant, whitening, anti-aging activities, and extremely low cytotoxicity. After fractional extraction, polyphenols and flavonoids were largely enriched in the ethyl acetate fraction (EaF), and total polyphenols and total flavonoids increased three- and fourfold, respectively, compared with CE. Especially, the rutin content increased 5.18-fold, quercetin increased 7.29-fold, and luteolin increased 10.58-fold. While chlorogenic acid and *p*-coumaric acid were mainly enriched in *n*-butanol fraction (NbF), and increased 2.1- and 2.75-fold than that in CE, respectively. The antioxidant, whitening and anti-aging activities of EaF are significantly higher than those of CE, especially the inhibition for hyaluronidase, elastase were greater than those of epigallocatechin gallate (EGCG), and its inhibitory effects on the tyrosinase and melanin content in B16F10 cells were stronger than those of kojic acid. NbF also showed lower IC_50_ values than EGCG against hyaluronidase and elastase. These results indicated that the Roman chamomile residue, especially the CE, EaF, and NbF, had excellent antioxidant, whitening, and anti-aging activities and could be a new natural raw material for use in functional cosmetic formulations.

## Graphical abstract

**Figure:**
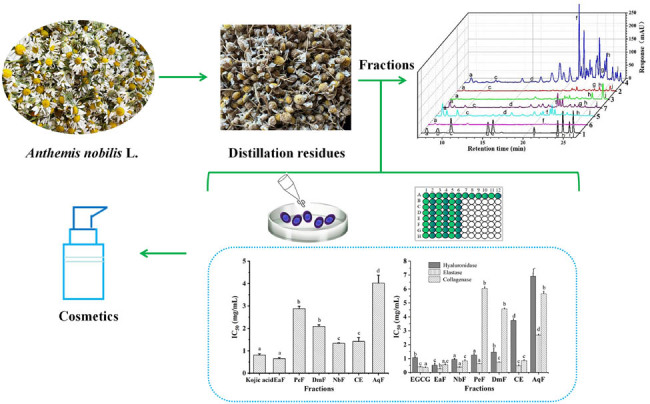

## 1 Introduction

Roman chamomile (*Chamaemelum nobile* (L.) All., *Anthemis nobilis* L.) is a well-known aromatic plant. It is an annual or perennial herb and belongs to the Compositae family [1]. Due to remarkable calming, antidepressant, anti-sensitive characteristics, essential oil of Roman chamomile is widely used in medicine, food, beverage, tobacco, spices, cosmetics, and so on [2,3]. However, a large amount of residues will generate when essential oil is extracted by steam distillation in industry due to its extremely low extraction rate [4,5]. These residues contain a lot of non-volatile and non-degradable components, which not only cause huge waste but also produce environmental pollution [6,7]. It is very important to investigate their composition and efficacy for recycling utilization and solving the pollution problem caused by improper disposal.

Besides essential oil, Roman chamomile contains abundant polyphenols and flavonoids, for example, apigenin, luteolin, rutin, quercetin, coumarin, caffeic acid, ferrocyanic acid, dibenzoic acid, pentanol, isobutanol, and other components. After the essential oil is extracted from Roman chamomile, these non-degradable polyphenols and flavonoids may be retained in the residue. The extract of Roman chamomile often displays excellent anti-inflammatory properties [8] and is widely used as an anti-sensitive ingredient in cosmetics. However, plant polyphenols and flavonoids usually have excellent whitening and anti-aging properties [9,10]. With people paying increasing attention to the safety of cosmetic raw materials [11], the development and application of botanical cosmetic raw materials have become a hot issue in the industry. Among them, whitening and anti-aging products have a large market. Therefore, investigating the composition and anti-aging activity of the residue following Roman chamomile essential oil extraction holds significant importance for enhancing the recycling and utilization of such residues.

Tyrosinase is the key enzyme that promotes melanin formation, which is related to skin whitening [12,13], and the ability to inhibit tyrosinase is generally taken as a main index for evaluating the whitening activity of cosmetic raw materials. Elastin possesses the ability to scavenge free radicals, thereby delaying skin aging. Collagen is instrumental in facilitating the regeneration and repair of skin cells. Hyaluronic acid stimulates collagen synthesis, which enhances skin elasticity and firmness. Consequently, elastin, collagen, and hyaluronic acid are essential components in the process of skin anti-aging [14]. However, the elastase, collagenase, and hyaluronidase will induce the degradation of collagen, elastin, and hyaluronic acid, respectively, and what was the main reasons for the formation of wrinkles and skin relaxation [15]. Therefore, the main test indicators for the anti-aging performance of cosmetic products are the inhibition effects on elastase, collagenase, and hyaluronidase in the field of cosmetics. In addition, another important aspect of skin anti-aging is antioxidants.

In the reported studies, more attention had been paid to the antibacterial effects of Roman chamomile oil [16,17]. And the phenolic components of flowers, which are unextracted by distillation, exhibit various medicinal properties, including anti-inflammatory, anti-cancer, and symptomatic relief of the common cold [18,19]. Roman chamomile extracts were increasingly utilized in the functional food industry as a natural alternative or supplement to synthetic ingredients [20]. Reports have shown that plants of the Compositae family have market potential as functional foods [21]. A previous study had shown that the solid residue obtained after extracting essential oil from plants could be used as a potential source of phenolics/antioxidants [22]. The components of the distillation residues would show significant changes when compared to Roman chamomile flowers [23], which will affect efficacy. To resolve the resource issue of residues and achieve low-carbon and zero-waste, the components of distillation residues are effectively recycled, which is combined with extraction technology. Thus, in this article, the components of distillation residues of Roman chamomile were analyzed, and its anti-oxidation, anti-aging, and whitening activities were evaluated. It would provide a reference for the utilization of the aromatic plant residues in cosmetics and the formation of a sustainable and complete production process.

## 2 Materials and methods

### 2.1 Plant materials and reagents

The flower parts of the Roman chamomile plant were produced in the Jiamusi region 46°48′35″N of latitude and 130°22′08″E of longitude (Heilongjiang Province, China) and picked. Plant material was identified by Dr. Zhong Wang of the Department of Intelligent Agriculture, College of Biology and Agriculture, Jiamusi University, Heilongjiang, China. The melanoma (B16F10) cells were purchased from the Cell Bank of the Chinese Academy of Sciences (Shanghai, China). 1,2-Diphenyl-2-picrylhydrazyl (DPPH) was purchased from MacLin Biochemical Technology Co., Ltd (Shanghai, China). Butylhydroxytoluene (BHT), epigallocatechin gallate (EGCG), *N*-[3-(2-furyl) acryloyl]-leucine glycine proline alanine (FALGPA), hyaluronic acid, hyaluronidase, elastase, and collagenase were purchased from Sigma Aldrich Trading Co., Ltd (Shanghai, China). All other chemical reagents were of analytical grade or above.

### 2.2 Extraction and fractionation

The selection of extractants was analyzed and determined based on the literature [24]. The residues of Roman chamomile were obtained by extracting the essential oil from Roman chamomile flowers through steam distillation and were further extracted three times, each time using 50% ethanol solvent, the extraction temperature was 60°C, the liquid–solid ratio was 50 mL/g, the extraction duration was 1 h, and the pH value was 7.0 ± 0.2. The ethanol extract (CE) was obtained by mixing, filtering, and concentrating the extracted solution. The extractant was selected by referring to the methods that have been reported. The crude extract (CE) was redissolved in water, and petroleum ether, dichloromethane, ethyl acetate, and *n*-butanol were added successively for liquid-liquid extraction. After removing the organic solvent under vacuum conditions, the dry petroleum ether fraction (PeF), dichloromethane fraction (DmF), ethyl acetate fraction (EaF), *n*-butanol fraction (NbF), and the raffinate phase (AqF) were obtained.

### 2.3 Determination of the contents of total polyphenols (TPs) and total flavonoids (TFs)

The contents of TP were measured using a modified colorimetric Folin–Ciocalteu method [25]. Value is expressed as gallic acid equivalents of the dry weight of the extract.

The contents of TPs were calculated according to equation (1).

(1) \[\text{TP}\hspace{.5em}(\text{mg}\hspace{.5em}\text{GAE}/\text{g}\hspace{.5em}\text{DW})=\frac{C\times V}{M},]\]

where *V* is the total volume of the sample, *M* is the quality of the sample, and *C* is the TP concentration by the calibration equation calculated.

The contents of TF were measured by colorimetric analysis [26]. Value is expressed as rutin equivalents of the dry weight of the extract. The contents of TFs were calculated according to equation (2).

(2) \[\text{TF}\hspace{.5em}(\text{mg}\hspace{.5em}\text{RTE}/\text{mg}\hspace{.5em}\text{DW})=\frac{C\times V}{M},]\]

where *V* is the total volume of the sample, *M* is the quality of the sample, and *C* is the TF concentration by the calibration equation calculated.

### 2.4 High-performance liquid chromatography (HPLC) measurement

The extract samples were dissolved in methanol and filtered by a filter membrane of 0.22 μm. HPLC was conducted at 30°C with detection at 330 nm, and the Agilent TC-C18 (4.0 mm × 250 mm) was installed. The gradient elution was performed with a mixture of methanol and 0.01% formic acid at a 1.0 mL/min flow rate. The injection volume of extract samples was 10.0 μL every time.

### 2.5 Evaluation of extracts antioxidant activity

The extract’s antioxidant activity was evaluated by experiments to measure the diphenylpicrohydrazine radical (DPPH˙) scavenging activity, the superoxide anion radical ( \[{\text{O}}_{2}^{\cdot -}]\] ) scavenging activity, and the iron trivalent (Fe^3+^) reduction power. The method of DPPH˙ and \[{\text{O}}_{2}^{\cdot -}]\] scavenging activity experiment of extracts was developed from the literature [27] and modified. The method of Fe^3+^ reduction power experiment of extracts was developed from the literature [28] and modified. The IC_50_ value (half maximal inhibitory concentration) was defined as the sample concentration required to clear 50% of the DPPH˙ and \[{\text{O}}_{2}^{\cdot -}]\] .

### 2.6 Evaluation experiment of extracts anti-aging activity

#### 2.6.1 Anti-hyaluronidase activity experiment

The method of determination of anti-hyaluronidase activity was developed from the literature [29] and modified. Briefly, 10 μL of each sample, with a gradient spanning 0–2.0 mg/mL, was added to a 96-well plate. Subsequently, 20 μL of hyaluronidase solution with a concentration of 4–10 U/mL was added into each sample well, and the mixture was allowed to stand for 15 min at 37°C to incubate after mixing. Then, 20 μL of hyaluronic acid (0.3 mg/mL) solution was added to the mixture, which was incubated for 45 min at 37°C. Subsequently, 200 μL of acidic albumin solution, which contains 0.1% bovine serum albumin, was added to precipitate the undigested hyaluronic acid and acidic albumin. The mixture was measured for the absorbance at a wavelength of 600 nm after 10 min at 25°C. The positive control of the experiment was EGCG. The measurements were repeated three times.

The hyaluronidase inhibition was calculated according to equation (3):

(3) \[\text{Hyaluronidase}\hspace{.5em}\text{inhibition}\hspace{.5em}( \% )=\frac{A-B-D}{C-D}\times 100 \% .]\]

where *A* represents the absorbance of hyaluronic acid, hyaluronidase, samples, and acid albumin, *B* represents the absorbance without hyaluronic acid, *C* represents the absorbance without samples and hyaluronidase, and *D* represents the absorbance without samples.

### 2.7 Anti-elastase activity experiment

The method of determination experiment of anti-elastase activity was developed from the literature [30] and modified. The reaction substrate was *N*-succinyl-tri-alanine-*p*-nitroaniline (SANA), which was used to monitor the release of *p*-nitroaniline at a wavelength of 410 nm. Briefly, 20 μL samples (0–1 mg/mL), 25 μL SANA solution (1 mmol/L), and 100 μL Tris-HCl buffer solution (100 mmol/L) were mixed thoroughly in 96-well plates and preincubated for 10 min at 37°C. Then, 25 μL elastase solution (0.5 U/mL) was added, and the mixture was then incubated for 15 min at 37°C to incubate. The absorbance was measured at a wavelength of 410 nm. The positive control of the experiment was EGCG. The measurements were repeated three times.

The elastase inhibition was calculated according to equation (4):

(4) \[\text{Elastase}\hspace{.5em}\text{inhibition}\hspace{.5em}( \% )=\frac{A-(B-C)}{A}\times 100 \% .]\]

The method of determination experiment of anti-collagenase activity was developed from the literature [31] and modified. Briefly, 40 μL collagenase solution (2 U/mL), 20 μL samples (0–1 mg/mL), and 80 μL Tris-HCl buffer solution (50 mmol/L) were mixed thoroughly in 96-well plates and preincubated for 15 min at 37°C. Then 60 μL FALGPA solution (1.0 mmol/L) was added to react at 37°C for 20 min. The positive control of the experiment was EGCG. The absorbance was measured at a wavelength of 345 nm. The measurements were repeated three times.

The collagenase inhibition was calculated according to equation (5):

(5) \[\text{Collagenase}\hspace{.5em}\text{inhibition}\hspace{.5em}( \% )=\frac{A-(B-C)}{A}\times 100 \% .]\]

In equations (4) and (5), *A* represents the absorbance of the blank control without samples, *B* represents the absorbance of the samples, and *C* represents the absorbance of the samples without enzymes.

### 2.8 Cell culture and cytotoxicity assays

The cell viability experiment of B16F10 was detected by the 3-(4,5-dimethylthiazol-2-yl)-2,5-diphenyltetrazolium bromide (MTT) assay. It was inoculated with 3 × 10^3^ B16F10 cells per well of the 96-well plate. The cells were cultured at 37°C in 5% CO_2_ environment for 24 h. Then, the cells were treated with phosphate-buffered saline twice. 200 μL of drug-containing culture medium was added to the mixture, which was incubated for an additional 24 h. Twenty microliters of MTT reagent was added to the mixture, which was incubated for 4 h. Finally, the supernatant of the mixture was discarded, and then, 150 μL of dimethyl sulfoxide (DMSO) was added to dissolve the formazan products. The absorbance was measured at a wavelength of 492 nm. The measurements were repeated three times. Cell cytotoxicity was calculated according to equation (6):

(6) \[\text{Cell}\hspace{.5em}\text{cytotoxicity}\hspace{.5em}( \% )=\left(1-\frac{A}{B}\right)\times 100 \% .]\]

### 2.9 Determination of B16F10 cells’ melanin content and cellular tyrosinase activity

The method of determination experiment of B16F10 cells’ melanin content was developed from the literature [32] and modified. It was inoculated with 10^4^ B16F10 cells per well of the 96-well plate. The cells were incubated at 37°C in 5% CO_2_ environment for 24 h. Then, the cells were further cultured for 72 h with different concentrations of extract samples (0–1 mg/mL). After that, 0.8 mL of 1 mol/L NaOH solution containing 10% DMSO reagent was added to 96-well plates, and then, the cells were cracked at 80°C for 1 h. The absorbance was measured at a wavelength of 492 nm. The positive control of the experiment was Kojic acid. The measurements were repeated three times. The melanin content was calculated according to equation (7):

(7) \[\text{Melanin}\hspace{.5em}\text{content}\hspace{.5em}( \% )=\frac{A}{B}\times 100 \% .]\]

The method of determination of the cellular tyrosinase activity was developed from the literature [33] and modified. Briefly, B16F10 cells were inoculated in a 96-well plate and cultured for 24 h, and the cells were further cultured for 72 h with different concentrations of extract samples (0.2–200 µg/mL). And then, the cells were lysed. The lysates were centrifuged at 13,000 rpm for 15 min at 4°C. One hundred microliters of lysate (containing 40 μg protein) was mixed with 100 μL l-tyrosine solution (2 mmol/L) in a 96-well plate and incubated for 1 h at 37°C. The absorbance was measured at a wavelength of 492 nm. The positive control of the experiment was Kojic acid. The measurements were repeated three times. The cell tyrosinase activity was calculated according to equation (8):

(8) \[\text{Cell}\hspace{.5em}\text{tyrosinase}\hspace{.5em}\text{activity}\hspace{.5em}( \% )=\frac{A}{B}\times 100 \% .]\]

In equations (6)–(8), *A* represents the absorbance of samples and *B* represents the absorbance without samples.

### 2.10 Statistical analysis

The data were presented as mean ± SD of the three determinations, and the IC_50_ value (half maximal inhibitory concentration) was defined as the sample concentration necessary to inhibit 50% of the enzyme activity, which was calculated by SPSS 16.0. The significant difference was analyzed by Duncan’s multiple range test (*p* < 0.05) with SPSS 16.0 software.

## 3 Results and discussion

### 3.1 The component of different fractions of the residue extract

Polyphenols and flavonoids are important secondary metabolites in plants and often used as special active substances in the cosmetics industry [34,35]. The TP and TF contents in the different fractions of the residue extract are shown in Table 1. The TP and TF contents in CE were 0.098 ± 0.002 mg GAE/mg DW and 0.217 ± 0.251 mg RTE/mg DW, respectively. After the CE was further purified by liquid–liquid partition to afford five fractions, it was found that most of the polyphenols and flavonoids were enriched in EaF and NbF, especially in EaF, the contents of TP and TF increased three- to fourfold, respectively.

**Table 1:** The TP and TF contents in different fractions of the Roman chamomile residue extract

Fractions	TP content (mg GAE/mg DW), mean ± SD	TF content (mg RTE/mg DW), mean ± SD
CE	0.098 ± 0.002a	0.217 ± 0.251a
PeF	0.044 ± 0.005b	0.191 ± 0.008b
DmF	0.061 ± 0.009c	0.173 ± 0.007c
EaF	0.300 ± 0.016d	0.802 ± 0.009d
NbF	0.134 ± 0.005e	0.283 ± 0.013e
AqF	0.037 ± 0.006b	0.032 ± 0.005f

TP content: gallic acid equivalents, calculated by dry weight; TF content: rutin equivalents, calculated by dry weight; values within columns followed by the same letter (a–f) do not differ statistically at *p* < 0.05.

The main phenols and flavonoids in Roman chamomile, such as chlorogenic acid, caffeic acid, *p*-coumaric acid, rutin, quercetin, luteolin, and apigenin, were detected by HPLC, and their contents in the fractions of the residue are shown in Table 2. In this study, all the above compounds were retained within CE, and the content of rutin was the highest (30.31 ± 0.05 mg/g), followed by chlorogenic acid (9.43 ± 0.00 mg/g). It has been reported [36] that chlorogenic acid (0.013–0.084% w/w) was determined in dried extracts. After fractional extraction, most of the components were enriched in EaF; the contents of rutin, quercetin, and luteolin in EaF were 5.18-, 7.29-, and 10.58-fold higher than those in CE, respectively. The content of chlorogenic acid was mainly enriched in NbF, and increased 2.75-fold than that in CE. Seven compounds were detected in the distillation residue. Caffeic acid had been enriched by the extractants, and then, the content of caffeic acid of distillation residues was lower than that reported [37], which also indicated that distillation residues were valuable.

**Table 2:** The content of chemical components in different fractions of the Roman chamomile residue extract

Compound	Content (mg/g) mean ± SD					
	CE	DeF	EaF	NbF	AqF	PeF
Chlorogenic acid	9.43 ± 0.00	3.18 ± 0.01	11.86 ± 0.02	19.60 ± 0.02	2.56 ± 0.00	3.06 ± 0.01
Rutin	30.31 ± 0.05	—	157.06 ± 0.62	4.80 ± 0.12	3.80 ± 0.01	—
Caffeic acid	0.80 ± 0.00	—	2.38 ± 0.01	1.33 ± 0.00	—	0.92 ± 0.01
Quercetin	2.00 ± 0.01	—	14.58 ± 0.02	—	—	2.83 ± 0.02
Luteolin	2.48 ± 0.01	6.63 ± 0.05	26.24 ± 0.02	2.70 ± 0.01	—	3.32 ± 0.02
Apigenin	1.37 ± 0.01	9.75 ± 0.03	6.83 ± 0.04	0.56 ± 0.02	—	2.95 ± 0.01
*p*-Coumaric acid	0.60 ± 0.00	—	0.62 ± 0.00	1.65 ± 0.03	—	—

–, not determined.

### 3.2 Antioxidant activities experiment of different extracts

**Figure 1:**
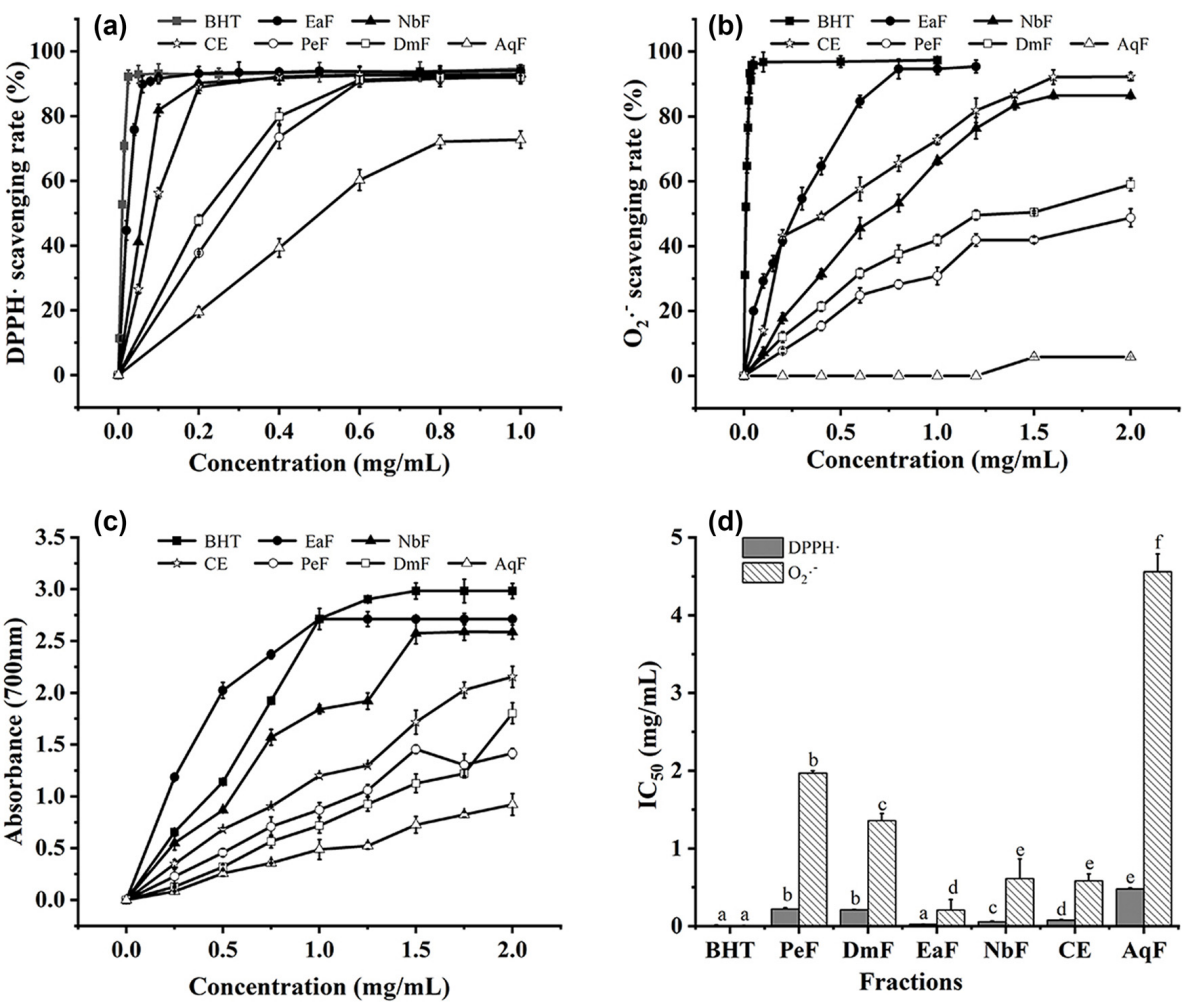
Different fractions of the Roman chamomile residue extract. (a) DPPH˙ scavenging activity, the concentrations of the CE and NbF: 0.05, 0.1, 0.2, 0.4, 0.6, 0.8, and 1.0 mg/mL, the concentrations of the EaF: 0.02, 0.04, 0.06, 0.08, 0.1, 0.2, 0.3, 0.4, 0.5, 0.75, and 1.0 mg/mL, and the concentrations of the PeF, DmF, and AqF: 0.2, 0.4, 0.6, 0.8, and 1.0 mg/mL; (b) \[{\text{O}}_{2}^{\cdot -}]\] scavenging activity, the concentrations of the CE and NbF: 0.1, 0.2, 0.4, 0.6, 0.8, 1.0, 1.2, 1.4, 1.6, and 2.0 mg/mL, the concentrations of the EaF: 0.05, 0.1, 0.15, 0.2, 0.3, 0.4, 0.6, 0.8, 1.0, and 1.2 mg/mL, and the concentrations of the PeF, DmF and AqF: 0.2, 0.4, 0.6, 0.8, 1.0, 1.2, 1.5, and 2.0 mg/mL; (c) Fe^3+^ reducing power, the concentrations of the CE, EaF, NbF, PeF, DmF, and AqF: 0.25, 0.5, 0.75, 1.0, 1.25, 1.5, 1.75, and 2.0 mg/mL; and (d) IC_50_ values of the DPPH˙ and \[{\text{O}}_{2}^{\cdot -}]\] scavenging activity. Values within columns followed by the same letter (a–f) do not differ statistically at *p* < 0.05.

The antioxidant activities of different extracts were assessed by DPPH˙ scavenging activity and \[{\text{O}}_{2}^{\cdot -}]\] scavenging activity and Fe^3+^ reduction power. The DPPH˙ scavenging rates of different fractions of the residue extract all increased in a dose-dependent manner. Except for AqF, the highest DPPH˙ scavenging rate of other fractions all reached more than 90%, which was close to BHT (Figure 1a). The highest scavenging activity of DPPH˙ was found in EaF and NbF. Literature [38] has shown that it has a relatively high antioxidant activity, which may be related to its high content of phenolic compounds.

Except for AqF, other fractions all presented dose-dependent scavenging activity for \[{\text{O}}_{2}^{\cdot -}]\] radical (Figure 1b), and among them, the scavenging rates of EaF, CE, and NbF all exceeded 90%, which were similar to BHT. EaF (IC_50_ = 0.207 ± 0.137 mg/mL) showed the most sensitive scavenging ability. CE (IC_50_ = 0.582 ± 0.093 mg/mL) also showed a preferable scavenging ability (Figure 1d). Studies had shown that the scavenging activity for \[{\text{O}}_{2}^{\cdot -}]\] radical (IC_50_ = 0.0360 mg/mL) was the fact that the more positively charged hydroxyl hydrogen atoms of flavonoids had, the more easily they were attacked by the negatively charged \[{\text{O}}_{2}^{\cdot -}]\] atoms, and the stronger the antioxidant activity [39].

All fractions exhibited a dose-dependent enhancement in the Fe^3+^ reducing power assay, and EaF and NbF showed the highest Fe^3+^ reducing power, close to BHT, followed by CE (Figure 1c).

In summary, CE of the residue showed strong antioxidant activity, especially in the DPPH˙ and \[{\text{O}}_{2}^{\cdot -}]\] scavenging abilities, close to BHT. After further fractionation, the EaF showed stronger antioxidant activity than CE. HPLC detection found that compared with CE, quercetin and rutin were highly enriched in EaF, and their contents were much higher than those in CE, which was the main reason why all indicators of antioxidant activity of EaF were higher than those of CE. This result was similar to the previously reported, which showed that quercetin has the highest antioxidant activity, followed by gallic acid, quercitrin, and rutin [40].

### 3.3 Anti-aging activity experiment of different extracts

**Figure 2:**
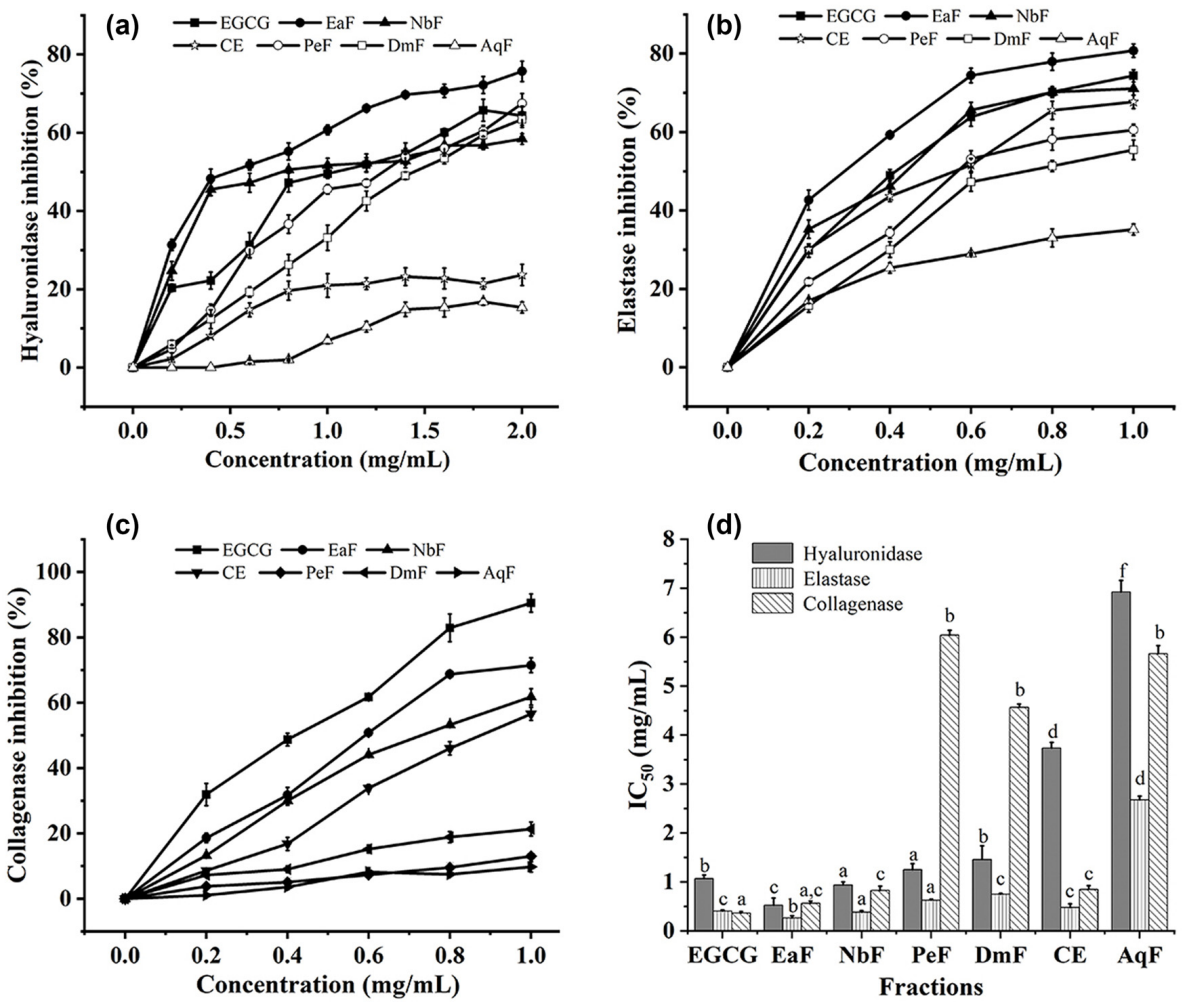
The inhibition of different fractions of the Roman chamomile residue extract on (a) hyaluronidase, the concentrations of the fractions: 0.2, 0.4, 0.6, 0.8, 1.0, 1.2, 1.4, 1.6, 1.8, and 2.0 mg/mL; (b) elastase, the concentrations of the fractions: 0.2, 0.4, 0.6, 0.8, and 1.0 mg/mL; (c) collagenase, the concentrations of the fractions: 0.2, 0.4, 0.6, 0.8, and 1.0 mg/mL; and (d) the IC_50_ values of the hyaluronidase, elastase, and collagenase inhibition activity. Values within columns followed by the same letter (a–f) do not differ statistically at *p* < 0.05.

The anti-aging activity of the Roman chamomile residue extracts was determined by hyaluronidase, elastase, and collagenase inhibition assays, and the inhibition of different fractions of residue extract on hyaluronidase, elastase, and collagenase is shown in Figure 2.

EaF, PeF, DmF, and NbF showed excellent inhibitory activity of hyaluronidase in a dose-dependent manner (Figure 2a). The highest inhibition rates of hyaluronidase by PeF, DmF, and NbF were around 60%. EaF was much higher than EGCG, which was close to 80%. The EaF (IC_50_ = 0.524 ± 0.146 mg/mL) also displayed the most sensitive inhibitory activity for hyaluronidase (Figure 2d).

Except for AqF, all other fractions showed preferable inhibitory ability against elastase in a dose-dependent manner, especially EaF was nearly 80% (Figure 2b). Literature reported that among the extracts obtained with *n*-butanol, *n*-butane, and ethyl acetate, the ethyl acetate extract has the best anti-elastase activity with IC_50_ at 11.7 mg/mL [41]. This study also showed that EaF (IC_50_ = 0.264 ± 0.044 mg/mL) exhibited the highest inhibitory activity against elastase (Figure 2d). The CE (IC_50_ = 0.476 ± 0.076 mg/mL) was close to EGCG and also displayed a good inhibitory activity on elastase (Figure 2d).

EaF, NbF, and CE showed better inhibitory activity for collagenase in a dose-dependent manner. The inhibition rates of all extracts were lower than those of the positive controls, but the inhibition rates of the EaF were also nearly 70% (Figure 2c). This was similar to the reported result [42], which showed that when the concentration of ethyl acetate extract is 1,000 μg/mL, the inhibition rate of collagenase was 75.14 ± 1.20%.

In a word, the EaF, NbF, and CE of the residue showed excellent anti-aging activity, especially their inhibitory activities against hyaluronidase and elastase were close to EGCG, which had great development prospects in anti-aging cosmetics.

### 3.4 Cytotoxicity experiment of different extracts

**Figure 3:**
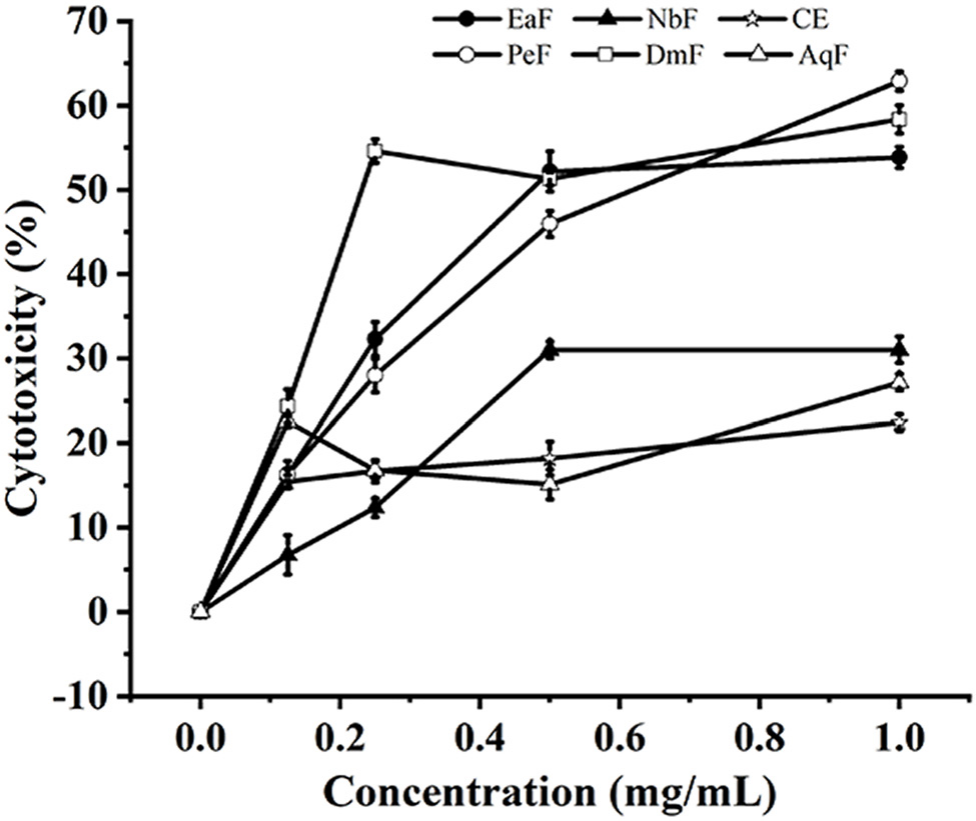
Cytotoxicity of different fractions of the Roman chamomile residue extract on the B16F10 cells. The concentrations of the fractions: 0.03, 0.06, 0.125, 0.25, 0.5, and 1.0 mg/mL, cell concentration: 3 × 10^3^ cells/well.

The cytotoxicity of different fractions of the Roman chamomile residue extract was investigated, as shown in Figure 3. Among all fractions, NbF, CE, and AqF showed extremely low cytotoxicity (below 30%) for B16F10 cells. In particular, the CE displayed the lowest cytotoxicity, which remained below 20%, which provided a guarantee for the application of the CE of Roman chamomile residue. The EaF, PeF, and DmF showed strong cytotoxicity.

### 3.5 Whitening activity experiment of different extracts

**Figure 4:**
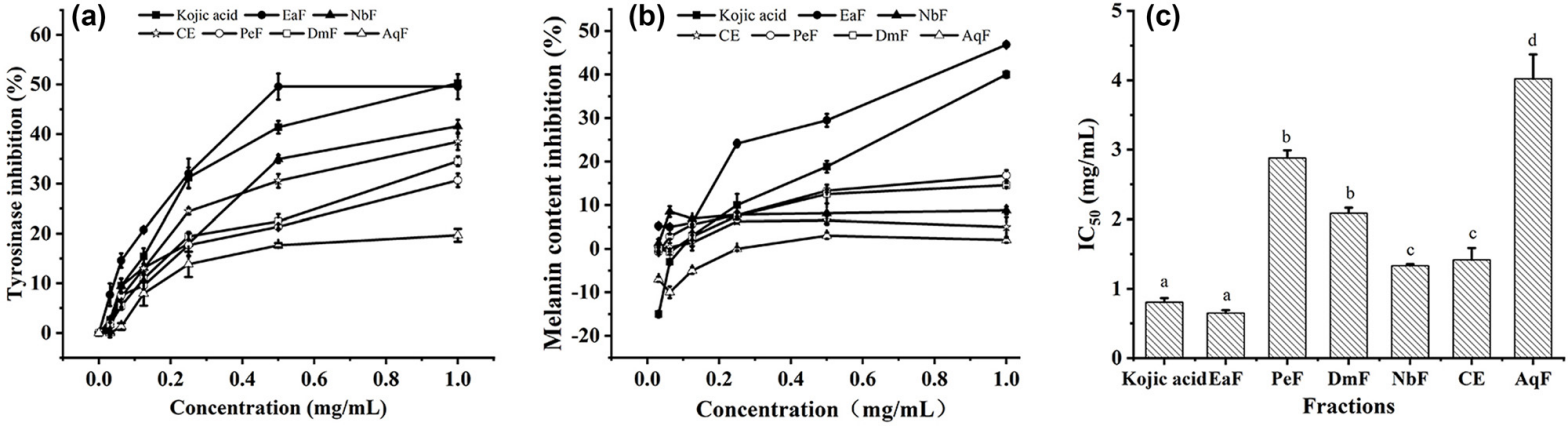
The different extracts of the Roman chamomile distillation residue for tyrosinase activity (a) and melanin content (b) inhibitory of B16F10 cells. Cell concentration: 3 × 10^3^ cells/well, the concentrations of the extracts: 0.03, 0.06, 0.125, 0.25, 0.5, and 1.0 mg/mL. (c) The IC_50_ values of the tyrosinase inhibition activity of B16F10 cells. Values within columns followed by the same letter (a–f) do not differ statistically at *p* < 0.05.

The different extracts of the Roman chamomile distillation residue for tyrosinase activity and melanin production inhibitory of B16F10 cells are shown in Figure 4. The report had all fractions exhibiting tyrosinase activity inhibitory of B16F10 cells at a dose-dependent manner. Particularly, the EaF (IC_50_ = 0.652 ± 0.427 mg/mL) showed that the highest inhibition rate was nearly 50%. The CE (IC_50_ = 1.417 ± 0.349 mg/mL) also showed good inhibitory activity on tyrosinase. The obtained results [43] showed how all extracts inhibited the tyrosinase enzyme in a range from 56.15 to 70.36 µg/mL in terms of IC_50_ values. Rinthong et al. [44] had proved that the highest tyrosinase inhibitory activity (IC_50_ = 0.7634 ± 0.01 mg/mL) was recorded in the extracts. It indicated that the research results were reliable, which compared with the reported results. Reports have shown that it might be that extracts non-competitively inhibit mushroom tyrosinase, or that extracts were oxidized, which reduced melanin formation [45]. The results indicated that the EaF and CE could have some whitening activity.

## 4 Conclusions

For the cosmetics industry, Roman chamomile distillation residue is a low-cost source. Through the hierarchical extraction and recycling of Roman chamomile residue components, the CE presented extremely low cytotoxicity and good antioxidant, whitening, and anti-aging activities. After enrichment with ethyl acetate, the antioxidant, whitening, and anti-aging activities of EaF were significantly improved, and within the safe dose range, it showed more activities than the positive controls, which was related to a large amount of enriched polyphenols and flavonoids. The CE and EaF can be new natural raw materials for cosmetics. The reuse of Roman chamomile distillation residue can establish a circular economy and achieve environmentally sustainable development.

## Supplementary Material

Supplementary Figure
