# Supplementary material for: Phytochemicals of Roman chamomile: Antioxidant, anti-aging, and whitening activities of distillation residues
Source: Open Life Sci. 2025 Oct 13;20(1):20251177. doi: 10.1515/biol-2025-1177 (PMC12552861; doi:10.1515/biol-2025-1177)
Supplement: Supplementary Figure [file biol-2025-1177-sm.pdf]

Supplementary material

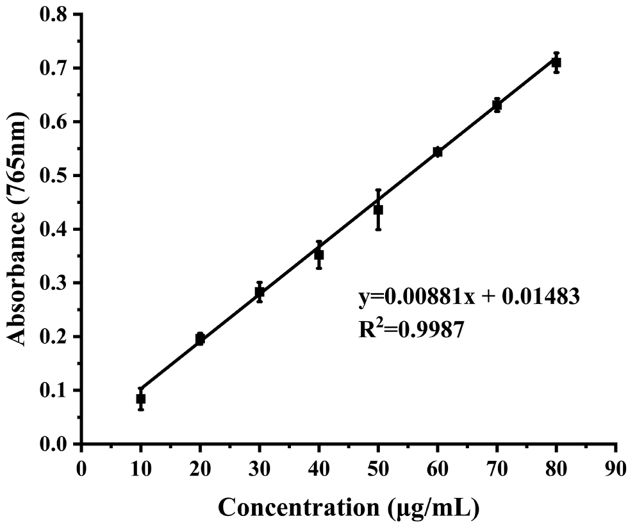

Figure S1: The standard curve of gallic acid. Values are means ± standard deviation of three determinations.

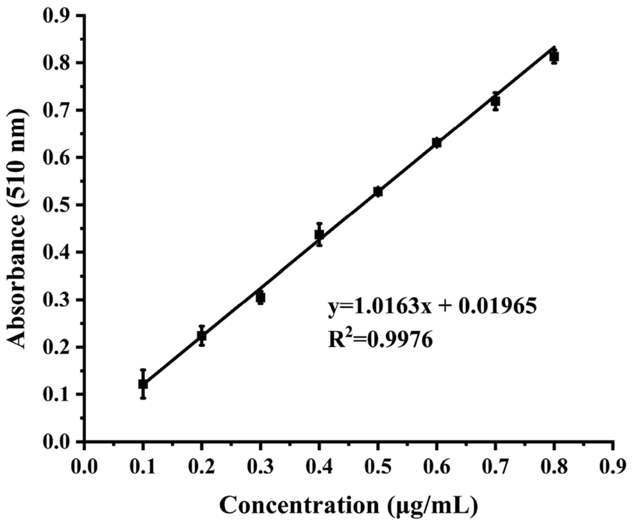

Figure S2: The standard curve of rutin. Values are means ± standard deviation of three determinations.

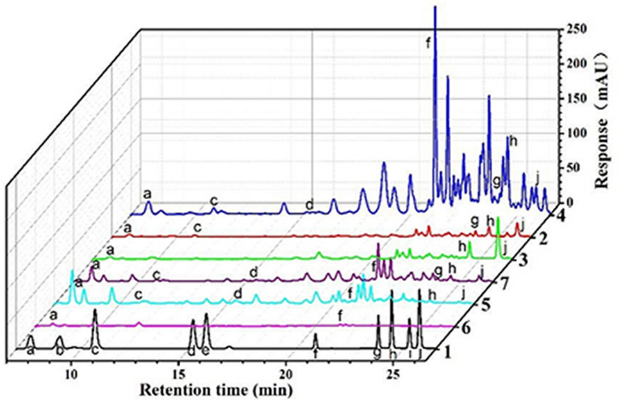

Figure S3: The chemical components in different fractions of the Roman chamomile residue extract by HPLC. 1→7: Mixed standard solution (25 μg/mL), the petroleum ether fraction, the dichloromethane fraction, the ethyl acetate fraction, the n-butanol fraction, the the aqueous fraction, the crude ethanolic extract. a→j: Chlorogenic acid, cynarin, caffeic acid, *p*-coumaric acid, ferulic acid, rutin, quercetin, luteolin, kaempferol, apigenin.
